# Effects of robot-assisted gait training in patients with Parkinson’s disease: study protocol for a randomized controlled trial

**DOI:** 10.1186/s13063-018-3123-4

**Published:** 2019-01-07

**Authors:** Min-Gu Kang, Seo Jung Yun, Hyun Iee Shin, Eunkyung Kim, Hyun Haeng Lee, Byung-Mo Oh, Han Gil Seo

**Affiliations:** 1 Department of Rehabilitation Medicine, Seoul National University Hospital, 101, Daehak-ro, Jongno-gu, Seoul, Republic of Korea; 2 Department of Rehabilitation Medicine, Konkuk University Medical Center, Seoul, Republic of Korea

**Keywords:** Gait, Parkinson disease, Exoskeleton device, Rehabilitation, Neuroimaging

## Abstract

**Background:**

Robot-assisted gait training (RAGT) was developed to restore gait function by promoting neuroplasticity through repetitive locomotor training and has been utilized in gait training. However, contradictory outcomes of RAGT have been reported for patients with Parkinson’s disease (PD). In addition, the mechanism of the RAGT treatment effect is still unknown. This study aims to investigate the effects of RAGT on gait velocity in patients with PD and to unveil the mechanisms of these effects.

**Methods:**

This is a prospective, single-blind, single-center, randomized controlled trial. Eligible participants will be randomly allocated to: 1) a Walkbot-S™ RAGT group or 2) a treadmill training group. The participants will receive three 45-min sessions of each intervention per week for 4 weeks. Gait speed during RAGT will be targeted to the maximal speed depending on the participant’s height; the same principle will be applied to the treadmill training group to match the training intensity. The primary outcome measure is gait speed measured by the 10-Meter Walk Test at a comfortable pace under single-task conditions. Secondary outcomes include dual-task interference, the Berg Balance Scale, Timed Up and Go test, the Korean version of the Falls Efficacy Scale-International, New Freezing of Gait Questionnaire, Movement Disorder Society-sponsored revision of the Unified Parkinson’s Disease Rating Scale, and functional connectivity measured by resting-state functional magnetic resonance imaging. Baseline assessments (T0) will be conducted to acquire clinical characteristics and outcome measure values before the intervention. Postintervention assessments (T1) will compare immediate efficacies within 3 days after the intervention. Follow-up assessments (T2) will be conducted 1 month after the intervention. Considering an alpha of 0.05 and a power of 80%, the total number of participants to be recruited is 44.

**Discussion:**

This study will reveal the effect of RAGT using an exoskeletal robot, not only on gait speed, but also on gait automaticity, balance function, fall risk, quality of life, and disease severity. In addition, the study will shed new light on the mechanism of the RAGT effect by evaluating changes in gait automaticity and brain functional networks.

**Trial registration:**

ClinicalTrials.gov, NCT03490578. Registered on 21 March 2018.

**Electronic supplementary material:**

The online version of this article (10.1186/s13063-018-3123-4) contains supplementary material, which is available to authorized users.

## Background

Robot-assisted gait training (RAGT) was developed to restore gait function by promoting neuroplasticity through repetitive locomotor training [1]. Exoskeletal and end-effector robots have been commercialized and utilized in gait rehabilitation [2]. In the area of brain diseases, most clinical studies have been conducted on stroke patients. According to a recent systematic review, machine- and robot-assisted gait training improved walking independency in subacute stroke patients who could not walk by themselves [3]. The American Heart Association/American Stroke Association (AHA/ASA) guideline for adult stroke rehabilitation recommended RAGT to improve motor function and mobility with a class IIb level of evidence A [4]. However, there is still a lack of studies of RAGT in brain diseases other than stroke, and clear guidelines are not provided.

Parkinson’s disease (PD) is a neurodegenerative disorder presenting with resting tremor, rigidity, bradykinesia, postural instability, and gait disturbance. Gait disturbance is one of the most disabling symptoms of PD [5]. Reduced step height, short stride, slow gait, and freezing of gait are common gait problems in patients with PD. Although pharmacological treatment has been the mainstay of PD management, most patients experience functional aggravation despite optimal medication [6]. Deep brain stimulation, a surgical intervention to treat PD, has become an acceptable treatment option. However, because of the risks and complications of the procedure, it has been limited to some advanced patients with medically refractory symptoms [7]. So far, no cure for PD has been developed. Therefore, rehabilitation has been an essential element in maintaining the maximum level of mobility and independence in patients with PD. For gait rehabilitation in PD, treadmill training has been a common approach. Thus, conventional treadmill training was selected as a comparator in this study protocol.

A major impairment causing gait disturbance in PD is the hypokinesia of gait [8]. Gait hypokinesia is associated with reduced stride length and step height, decreased cadence, and prolonged double limb support. It is one of the most disturbing symptoms affecting quality of life in PD [9] and can be quantified as the gait velocity. Decreased gait automaticity is another important cause of gait disturbance in PD [10]. Once a skilled movement has been learned, the movement becomes automatic and does not require much conscious endeavor or attention. Gait automaticity refers to the ability to walk without cognitive effort or attention [11]. In patients with PD, loss of dopamine in the sensorimotor territories of the basal ganglia disturbs habitual control [12], so that gait automaticity is decreased and patients have to rely on attentional resources or make a conscious effort to walk. Dual-task performance demands greater attentional cost and cognitive effort, and therefore gait automaticity can be quantified as the dual-task interference. For these reasons, this study will focus on the change in gait velocity and dual-task interference between before and after the intervention.

There are studies of the effects of RAGT on gait velocity with better outcomes for RAGT than the control group [13–16], as well as insignificant outcomes [17–19]. Some studies on PD have suggested that RAGT might improve freezing of gait, postural instability, and gait performance [13–16, 20]; however, several studies have reported that RAGT is not superior to conventional therapy for improving postural instability and gait performance [17, 18, 21]. Due to the contradictory results, a well-designed study is needed to clarify the effect of RAGT on gait function in PD. Impaired automaticity leads to gait disturbance and falls in PD, especially in situations when the patients conduct secondary tasks in parallel with gait. So far, there are no studies that have investigated the effect of RAGT on gait automaticity in PD; therefore, a further study is needed to reveal this effect.

There appears to be an association between brain networks and gait function in PD. Functional connectivity has been reported to be decreased even in the early stages of PD [22, 23], and freezing of gait might be a consequence of alterations in brain functional networks [24, 25]. RAGT was suggested to improve gait recovery by promoting brain plasticity [26]. Therefore, there is a need to study changes in functional connectivity after RAGT in PD. In this study, resting-state functional magnetic resonance imaging (MRI) will be conducted to discover significant changes in functional connectivity and compare the functional networks of the brain before and after the intervention.

The primary objective of this study is the verification of the hypothesis that RAGT can produce greater improvement, compared with conventional treadmill training, in gait speed at a comfortable pace. Secondarily, this study aims to determine whether gait automaticity, balance, mobility, fall risk, and disease severity are improved by RAGT. Thirdly, this trial will shed new light on the mechanism of the RAGT treatment effect by investigating dual-task interference and functional connectivity before and after the intervention.

## Methods

### Trial design

**Fig. 1:**
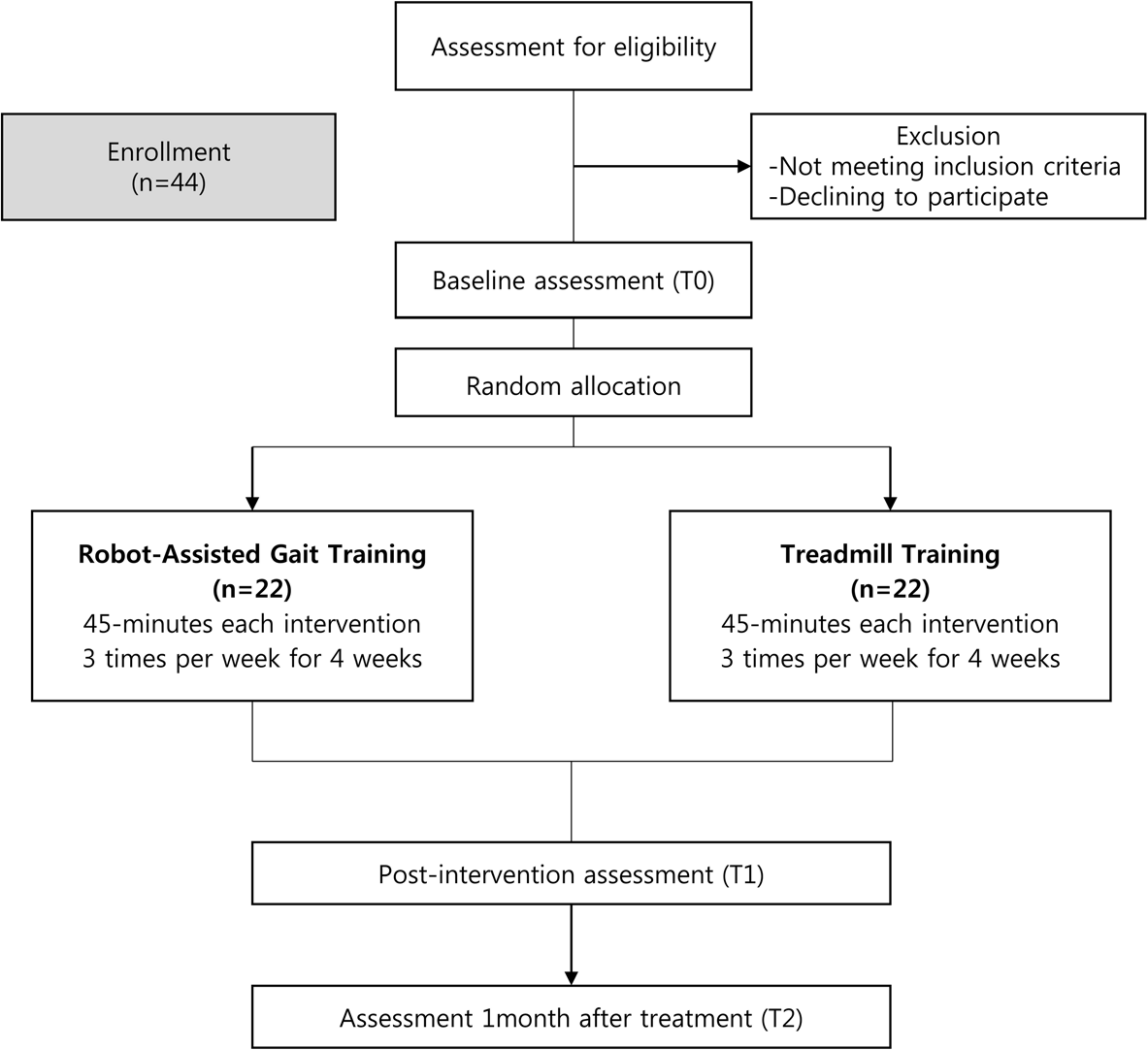
Consolidated Standards of Reporting Trials (CONSORT) flow diagram

This is a prospective, single-blind (assessors), single-center, randomized controlled trial. Eligible participants will be randomly allocated to: 1) a RAGT group or 2) a treadmill training group. Figure 1 shows the Consolidated Standards of Reporting Trials (CONSORT) flow diagram for this study. Baseline assessments (T0) will be conducted to acquire clinical characteristics and values of the primary and secondary outcome measures before the intervention. Postintervention assessments (T1) to compare the immediate efficacy between the two groups will be performed within 3 days after the intervention. Follow-up assessments (T2) will be conducted 1 month after the intervention. All procedures have been approved by the Institutional Review Board of the Seoul National University Hospital in accordance with good clinical practices, the Helsinki Declaration, and national regulations. The clinical trial was also approved by the Korean Ministry of Food and Drug Safety.

### Sample size

The primary outcome measure in this study is gait speed in postintervention assessments measured by the 10-m walk test (10MWT) at a comfortable pace under single-task conditions. Previous studies, in which similar populations and intervention protocols were employed, have reported that the minimal detectable change in comfortable gait speed measured by the 10MWT was 0.18 m/s [27] and the standard deviation was 0.08–0.20 m/s [13, 17]. Based on those findings, we consider 0.18 m/s as a clinically significant improvement in gait speed and 0.20 m/s as an acceptable standard deviation. Consequently, 20 participants per group will be required to achieve 80% power with a two-tailed α of 0.05, as determined by [28]:

$$ N=\frac{2{SD}^2{\left({Z}_{\alpha }+{Z}_{\beta}\right)}^2}{\triangle^2} $$

With an expected drop-out rate of 10%, a total of 44 participants should be recruited, 22 for each group, to establish the relative efficacy of each intervention in improving gait velocity.

### Participants

In total, 44 participants will be recruited from the Department of Rehabilitation of the Seoul National University Hospital (Seoul, Republic of Korea). Approved trial posters including the inclusion, exclusion criteria, and contact details of researchers will be displayed in appropriate clinics.

The inclusion criteria are:

- Clinically diagnosed as idiopathic PD
- Men or women aged ≥ 18 years
- Hoehn-Yahr stage 2.5 or 3
- Mini-Mental State Examination (MMSE) score ≥ 24

The exclusion criteria include:

- Severe dyskinesia or severe on-off phenomenon
- Plan to adjust medication at the time of screening
- Sensory dysfunction in lower extremities
- Vestibular disease or benign paroxysmal positional vertigo
- Severe medical problems such as cardiovascular diseases
- Other neurological or orthopedic disorders affecting lower extremities

Participants will be individually consented to participate in the entire study. The Principal Investigator and clinical research coordinator will obtain the written informed consent from all participants.

### Randomization and blinding

Randomization and allocation will be performed using computer-generated block randomization with a block size of 4 and 6. An independent researcher who is not in contact with any patient will perform the randomized allocation. The ratio between the RAGT and treadmill training groups will be 1:1. Details of the allocated group will be given in sequentially numbered, opaque, sealed envelopes to the research assistants in sequential order. The principal investigator, outcome assessors, and data analysts will be blinded to the group allocations of the participants until statistical analysis.

### Interventions

**Fig. 2:**
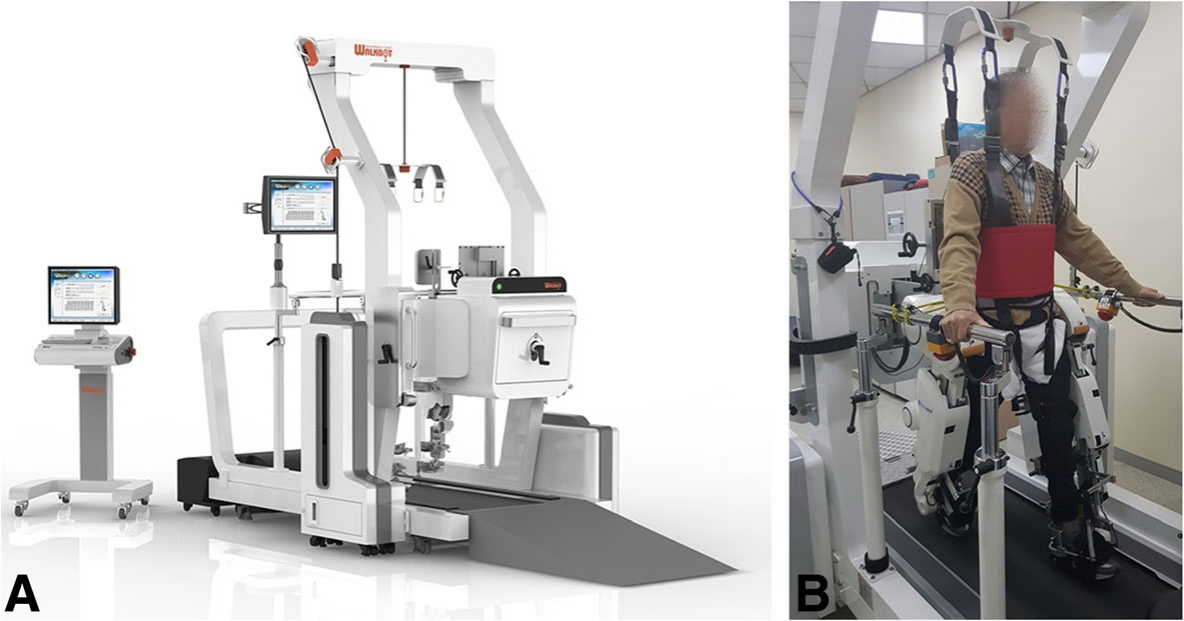
**a** The Walkbot-S™, an exoskeletal-type gait training robot; **b** a patient participating in gait training using Walkbot-S™

Participants in the RAGT group will receive gait training with the Walkbot-S™ (P&S Mechanics, Seoul, Korea) for 12 sessions (Fig. 2). Training is preceded by fitting and adapting the equipment to the participant. In the first training session, RAGT starts at the reference velocity depending on the participant’s height (Table 1) and reaches 80% of the maximal velocity at the end. Starting with the third training session, the initial gait velocity is increased and the final velocity becomes the maximal velocity depending on the participant’s height. Starting with the seventh training session, training velocity reaches the maximal velocity after 10 min. Table 2 shows an example of a training protocol for a participant whose height is 160 cm.

**Table 1:** Walkbot-S™ reference and maximal velocities depending on height

	Height						
	140 cm	150 cm	160 cm	170 cm	180 cm	190 cm	200 cm
Reference velocity (m/s)	1.0	1.1	1.2	1.3	1.4	1.5	1.6
Maximal velocity (m/s)	1.8	2.0	2.2	2.4	2.6	2.8	3.0

**Table 2:** Example of a training velocity protocol for a 160-cm tall participant

	No. of minutes						
	0–5	5–10	10–15	15–20	20–25	25–30	30
Training session no.	Velocity, m/s						
1	1.2	1.2	1.4	1.4	1.7	1.7	1.7
2	1.2	1.2	1.4	1.4	1.7	1.7	1.7
3	1.4	1.4	1.8	1.8	2.2	2.2	2.2
4	1.4	1.4	1.8	1.8	2.2	2.2	2.2
5	1.7	1.7	2	2	2.2	2.2	2.2
6	1.7	1.7	2	2	2.2	2.2	2.2
7	1.9	1.9	2.2	2.2	2.2	2.2	2.2
8	1.9	1.9	2.2	2.2	2.2	2.2	2.2
9	1.9	1.9	2.2	2.2	2.2	2.2	2.2
10	1.9	1.9	2.2	2.2	2.2	2.2	2.2
11	1.9	1.9	2.2	2.2	2.2	2.2	2.2
12	1.9	1.9	2.2	2.2	2.2	2.2	2.2

During gait training, the robot provides an auditory cue at the toe-off phase to improve gait rhythmicity. Moreover, the robot provides a visual feedback by displaying a warning message on the screen when the torque exceeds a preset target range, which induces active hip flexion. The cue and feedback are designed to induce active participation in the training. The treatment time per session is 45 min, including don-and-doff, warm-up, and cool-down. The actual RAGT time is 30 min. A total of 12 sessions are provided over the course of 4 weeks.

Participants in the treadmill training group receive gait training on the treadmill. The same velocity protocol as in the RAGT group is applied to the treadmill training group to match the training intensity. A physical therapist provides appropriate visual and auditory instructions to allow the patient to participate in gait training actively. The treatment time per session is 45 min, including warm-up and cool-down. The actual treadmill training time is 30 min. A total of 12 sessions are provided over the course of 4 weeks.

The participants are contacted by phone and mobile text message 1 day before each session to improve adherence. During the clinical trial, participants are prohibited from changing the regimen of dopaminergic medication but they are permitted to keep the previous rehabilitation therapy that was already received. The participation can be discontinued due to the occurrence of serious adverse events or at the participant’s request. Fidelity of the intervention will be assessed through documenting the number and duration of sessions and the minimal and maximal velocity of each session delivered.

### Outcome measures

The primary outcome measure is gait speed measured by the 10MWT at a comfortable pace under single-task conditions [29]. The 10MWT is a common measure to evaluate gait velocity and highly reliable for assessing gait speed in patients with PD [29].

The secondary outcomes include dual-task interference [30], the Berg Balance Scale (BBS) [31], Timed Up and Go (TUG) test [32], the Korean version of the Falls Efficacy Scale-International (KFES-I) [33, 34], the New Freezing of Gait Questionnaire (NFOG-Q) [35], and the Movement Disorder Society-sponsored revision of the Unified Parkinson’s Disease Rating Scale (MDS-UPDRS) [36].

Dual-task interference, an indicator of gait automaticity, is defined by the difference between dual- and single-task performance [37]. To evaluate dual-task performance, cognitive dual-task walking [38] will be measured while performing the Wechsler Forward Digit Span [39], and physical dual-task walking will be measured while carrying a tray with two cups of water [40, 41]. The BBS measures balance function during sitting, standing, and changing positions. It consists of 14 items and the total score ranges from 0 to 56 [42]. The tool is clinically valid in PD patients [31]. The TUG test is a reliable measure for mobility in the elderly. During the test, patients stand up from a chair, walk for 3 m, turn, walk back, and sit down. It is also a highly reliable tool to assess mobility in PD [32]. KFES-I is a valid method to assess fear of falling in the elderly [33]. It is a self-reported measure of concerns about falls during activities of daily living. It consists of 16 items scored on a four-point scale [34]. NFOG-Q is a freezing of gait (FOG) questionnaire which assesses FOG severity and gait disturbances. It is a reliable tool to measure the FOG and the functional impact in patients with PD [35]. MDS-UPDRS, a clinical rating scale for PD, contains four parts [36]. A total of 50 questions rate disability or impairment on a scale of 0–4.

**Fig. 3:**
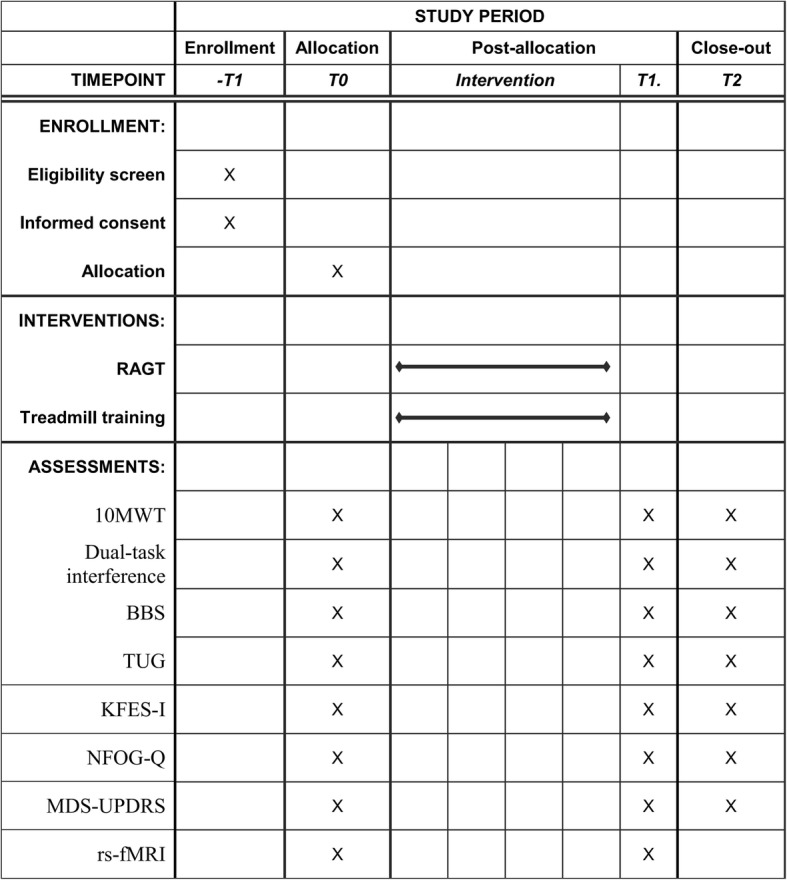
Study schedule of enrollment, interventions, and assessments. 10MWT 10-m walk test, BBS Berg Balance Scale, KFES-I Korean version of the Falls Efficacy Scale-International, MDS-UPDRS Movement Disorder Society-sponsored revision of the Unified Parkinson’s Disease Rating Scale, NFOG-Q New Freezing of Gait Questionnaire, RAGT robot-assisted gait training, rs-fMRI resting-state functional magnetic resonance imaging, TUG Timed Up and Go

The 10MWT, BBS, and TUG test will be assessed by a physical therapist and the MDS-UPDRS will be administered by a licensed clinician. All assessors will be blinded to group assignment. Figure 3 shows the schedule for outcome measures evaluated at each visit.

### Functional MRI

Functional MRI will be employed to analyze functional connectivity before and after the intervention. Images will be obtained by a 3-T scanner (Siemens Magnetom TrioTim) using the following parameters: 116 volumes with a matrix size of 128 × 128 mm and a voxel size of 1.88 × 1.88 × 4.20 mm, TR = 3500 ms, TE = 30 ms, flip angle = 90°, and FOV = 240 × 240 mm. All participants will be instructed to remain still but awake with the eyes closed during the resting-state functional MRI scan.

### Data collection, management, and dissemination

Demographic data including age, sex, height, duration of disease, Hoehn-Yahr stage, MMSE score, and comorbidities will be obtained. Outcome measures at T0, T1, and T2 will be acquired as well. All data will be de-identified using an identification number to provide confidentiality and will be collected on paper case report forms and password-protected digital files. Paper case report forms and digital files will be stored in a locked cabinet in a locked office and on a password-protected computer in a locked office, respectively. An independent clinical research assistant will verify the data by review of medical records once a month to guarantee data quality including completeness and accuracy. Any adverse events, any untoward medical occurrence associated with the intervention of this study, will be recorded and reported to the Institutional Review Board and Korean Ministry of Food and Drug Safety (KMFDS). Additional medical procedures will be provided depending on the event. Trial management may be audited by the KMFDS which is independent of investigators at any time.

All authors will have access to the final dataset. Upon completion, trial results will be reported to the funders and submitted to peer-reviewed journals to communicate to participants, healthcare professionals, and the public. Authorship will follow guidelines recommended by the International Committee of Medical Journal Editors (ICMJE). Professional writers will not be employed.

### Statistical analysis

Data will be analyzed using the Statistical Package for the Social Sciences (SPSS) version 20. Statistical significance will be accepted when a *p* value is less than 0.05. The intention-to-treat (ITT) principle will be adopted in all analyses. All participants randomized to a group will remain in the group regardless of protocol violations or dropout. Last observation carried forward (LOCF) will be used to impute missing values. Per-protocol analyses will also be conducted. All participants from the ITT population without serious protocol violations will be included in the per-protocol population. Serious violations include exceptions to eligibility criteria and deviations from the treatment scheme such as not completing at least eight of the total of 12 sessions.

For the primary outcome analysis, the change between T0 and T1 in gait speed at a comfortable pace under single-task conditions will be analyzed using Student’s *t* test. Generalized estimating equation (GEE) models will be employed to identify differential changes of secondary outcome measures across multiple time points.

Functional connectivity in the whole brain area will be analyzed using the primary motor cortex as a seed region. Pearson’s correlation coefficient will be used to calculate functional connectivity. Comparisons of functional connectivity will be conducted using two sample *t* tests for between-group differences and paired *t* tests for within-group effects.

## Discussion

Robots have been increasingly used for gait rehabilitation, especially in patients with brain diseases such as stroke and multiple sclerosis [43, 44]. However, contradictory results have been reported by studies of RAGT in patients with PD. In addition, there are no clinical guidelines for RAGT in gait training for PD. Moreover, even in studies reporting gait improvement by RAGT in PD, the mechanism of the treatment effect remains elusive.

RAGT in this study will include a visual feedback and an auditory cue, which are known as effective strategies to treat locomotor impairments [45]. Feedback or cues were reported to improve gait performance in patients with PD [46], and an additional beneficial effect of cues with gait training has been suggested [47]. External cues appear to help patients with PD walk better by compensating for the impaired central drive for walking [48]. Another hypothesis is that external cues enable patients to utilize the intact premotor cortex, rather than the impaired basal ganglia/supplementary motor area circuit [49]. Therefore, RAGT including external cues or feedback is expected to improve gait performance in patients with PD.

This study will reveal the effect of RAGT using an exoskeletal robot, not only on comfortable gait speed, but also on dual-task interference, balance function, fall risk, and disease severity. In addition, the findings of the study will provide knowledge on the recovery of gait automaticity and brain functional connectivity following RAGT. We hope that the information acquired from this trial will advance our understanding of the mechanism of the RAGT treatment effect and improve clinical decision making when physiatrists consider gait training for patients with PD.

A limitation of this study is that it involves only one center. This might influence the generalizability of the results to other centers. Another limitation is that blinding of patients and therapists is not possible due to the definite different feature of the interventions.

In summary, this trial will compare the effects of RAGT with those of treadmill training on gait performance in patients with PD. The results of this study will provide clear evidence whether RAGT rather than treadmill training may be recommended for gait training in patients with PD.

### Trial status

This study was initiated in May 2018, and patient recruitment is ongoing. The trial will be completed in December 2019.

## Additional file

Additional file 1: Standard Protocol Items: Recommendations for Interventional Trials (SPIRIT) Checklist. (DOC 121 kb)

## Abbreviations

10MWT: 10-Meter walk test
AHA: American Heart Association
ASA: American Stroke Association
BBS: Berg Balance Scale
CONSORT: Consolidated Standards of Reporting Trials
GEE: Generalized estimating equation
ICMJE: International Committee of Medical Journal Editors
ITT: Intention-to-treat
KFES-I: Korean version of the Falls Efficacy Scale-International
KMFDS: Korean Ministry of Food and Drug Safety
LOCF: Last observation carried forward
MDS-UPDRS: Movement Disorder Society-sponsored revision of the Unified Parkinson’s Disease Rating Scale
MMSE: Mini-Mental State Examination
MRI: Magnetic resonance imaging
NFOG-Q: New Freezing of Gait Questionnaire
PD: Parkinson’s disease
RAGT: Robot-assisted gait training
TUG: Timed Up and Go
