# Correction to: Effects of robot-assisted gait training in patients with Parkinson’s disease: study protocol for a randomized controlled trial

**DOI:** 10.1186/s13063-020-04437-5

**Published:** 2020-05-27

**Authors:** Min-Gu Kang, Seo Jung Yun, Hyun Iee Shin, Eunkyung Kim, Hyun Haeng Lee, Byung-Mo Oh, Han Gil Seo

**Affiliations:** 1 Department of Rehabilitation Medicine, Seoul National University Hospital, 101, Daehak-ro, Jongno-gu, Seoul, South Korea; 2 Department of Rehabilitation Medicine, Konkuk University Medical Center, Seoul, South Korea

**Correction to: Trials (2019) 20:15**

10.1186/s13063-018-3123-4

Following publication of the original article [1], the authors identified an error in Tables 1 and 2. The unit of velocity should read ‘km/h’ instead of ‘m/s’.

**Table 1.** Walkbot-S™ reference and maximal velocities depending on height

	Height						
	140cm	150cm	160cm	170cm	180cm	190cm	200cm
Reference velocity (km/h)	1.0	1.1	1.2	1.3	1.4	1.5	1.6
Maximal velocity (km/h)	1.8	2.0	2.2	2.4	2.6	2.8	3.0

**Table 2.** Example of a training velocity protocol for a 160-cm-tall participant

	No. of minutes						
	0-5	5-10	10-15	15-20	20-25	25-30	30
Training session no.	Velocity, km/h						
1	1.2	1.2	1.4	1.4	1.7	1.7	1.7
2	1.2	1.2	1.4	1.4	1.7	1.7	1.7
3	1.4	1.4	1.8	1.8	2.2	2.2	2.2
4	1.4	1.4	1.8	1.8	2.2	2.2	2.2
5	1.7	1.7	2	2	2.2	2.2	2.2
6	1.7	1.7	2	2	2.2	2.2	2.2
7	1.9	1.9	2.2	2.2	2.2	2.2	2.2
8	1.9	1.9	2.2	2.2	2.2	2.2	2.2
9	1.9	1.9	2.2	2.2	2.2	2.2	2.2
10	1.9	1.9	2.2	2.2	2.2	2.2	2.2
11	1.9	1.9	2.2	2.2	2.2	2.2	2.2
12	1.9	1.9	2.2	2.2	2.2	2.2	2.2
